# The Ruminant Farm Systems Animal Module: A Biophysical Description of Animal Management

**DOI:** 10.3390/ani11051373

**Published:** 2021-05-12

**Authors:** Tayler L. Hansen, Manfei Li, Jinghui Li, Chris J. Vankerhove, Militsa A. Sotirova, Juan M. Tricarico, Victor E. Cabrera, Ermias Kebreab, Kristan F. Reed

**Affiliations:** 1 Department of Animal Science, Cornell University, Ithaca, NY 14850, USA; tlhansen@cornell.edu (T.L.H.); cjv47@cornell.edu (C.J.V.); mas769@cornell.edu (M.A.S.); 2 Department of Animal and Dairy Sciences, University of Wisconsin, Madison, WI 53706, USA; mli497@wisc.edu (M.L.); vcabrera@wisc.edu (V.E.C.); 3 Department of Animal Science, University of California, Davis, CA 95616, USA; jhuli@ucdavis.edu (J.L.); ekebreab@ucdavis.edu (E.K.); 4 Sustainability Research, Innovation Center for US Dairy, Rosemont, IL 60018, USA; juan.tricarico@dairy.org

**Keywords:** dairy management, Monte Carlo simulation, RuFaS

## Abstract

**Simple Summary:**

Consumers are increasingly concerned about the sustainable production of food, leading producers and scientists to evaluate farming practices that preserve environmental resources, provide adequate production, and are economically viable. However, there are challenges to synthesize these results and apply them on-farm in a holistic nature. Simulation modeling of farm systems, such as the dairy system, can allow producers, industry members, and policy makers to prioritize interventions that improve sustainable outcomes. We introduce the Animal Module of the Ruminant Farm Systems (RuFaS) model—a whole farm dairy system model—and describe its use to assess the environmental impact of improved feed efficiency in dairy cows. By decreasing the amount of feed intake required to produce the same amount of milk, the RuFaS model provides estimates of the reduction in feed use, enteric methane, and manure production.

**Abstract:**

Dairy production is an important source of nutrients in the global food supply, but environmental impacts are increasingly a concern of consumers, scientists, and policy-makers. Many decisions must be integrated to support sustainable production—which can be achieved using a simulation model. We provide an example of the Ruminant Farm Systems (RuFaS) model to assess changes in the dairy system related to altered animal feed efficiency. RuFaS is a whole-system farm simulation model that simulates the individual animal life cycle, production, and environmental impacts. We added a stochastic animal-level parameter to represent individual animal feed efficiency as a result of reduced residual feed intake and compared High (intake = 94% of expected) and Very High (intake = 88% of expected) efficiency levels with a Baseline scenario (intake = 100% of expected). As expected, the simulated total feed intake was reduced by 6 and 12% for the High and Very High efficiency scenarios, and the expected impact of these improved efficiencies on the greenhouse gas emissions from enteric methane and manure storage was a decrease of 4.6 and 9.3%, respectively.

## 1. Introduction

The FAO [1] predicts the world population will reach 9.8 billion people by 2050, requiring significant changes to food production to provide nutritious products to all people. Expanding populations with increased disposable income has led to a shift in dietary preferences, with emerging markets favoring animal products [2]. Animal products are important food, industrial, and health sources but are associated with increased environmental costs [3,4]. While some markets favor the production of more animal proteins, others are increasingly concerned about environmental impacts and sustainable production [5]. Sustainable production requires a systems approach to account for food security, environmental stewardship, and societal impacts [6].

**Figure 1:**
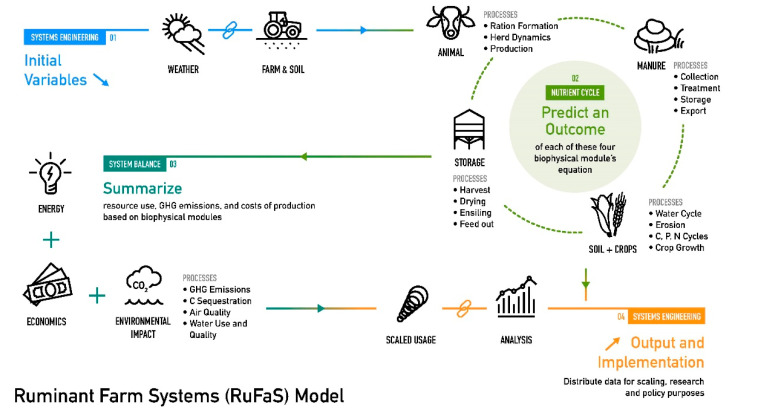
Conceptual diagram of the Ruminant Farm Systems Model.

Managing the need for sustainable food production requires a careful allocation of limited farm resources. One method to guide policy, support farm decisions, and evaluate novel technologies is the use of simulation models. The effect of implementing new technologies on-farm or changing production management can be evaluated on multiple outcomes, generally providing a more robust evaluation than traditional research experiments. Existing whole-farm models for dairy production include the Integrated Farm Systems Model [7], DairyMod [8], DyNoFlo [9], and SIMS(DAIRY) [10], but these models have limitations that prevent their wide-scale applicability in current and future scenarios. Incorporating vast amounts of data from farm records or adapting to new technologies is often impossible with existing models due to the model structure or restrictions in the model code. Thus, we saw the need to develop a new farm simulation model that can adapt to changing technologies and support sustainable dairy production [11]. The Ruminant Farm Systems Model (RuFaS, Figure 1) incorporates modern computer coding practices centered around clarity and adaptability to respond to evolving technologies in the dairy industry. RuFaS embraces the key characteristics for next-generation agricultural systems models described by Jones et al. [12]: “technological advances; open, harmonized data; transdisciplinary collaboration; modularity and interoperability; user-driven data and model development”.

Within the RuFaS framework, the environmental impacts of dairy production can be determined from each of the four main parts of a dairy farm: animals, manure, field, and feed storage. Previous life-cycle assessments (LCA) of dairy production have estimated the total environmental impacts of dairy production and the relative contributions of each part of the dairy farm and supply chain (e.g., [13,14]). Thoma et al. [13] attributed 73% of GHG emissions from US dairy production in 2008 to on-farm sources with 26, 34, and 33% of the on-farm emissions attributed to feed production, enteric methane, and manure emissions, respectively. The works by Capper et al. [15] and Capper and Cady [14] demonstrate the significant reductions in farm-gate GHG intensity (kg CO_2_ eq/kg milk) achieved in past decades by comparing LCA estimates of US dairy GHG footprints in 1944, 2007, and 2017.

Much of the reduction in GHG emissions per kg of milk is attributable to improvements in feed efficiency. In particular, Capper and Bauman [16] highlight the impact of increased milk production per cow that results in the dilution of maintenance effect, whereby the fixed cost (and GHG production) of maintaining an animal’s basic life function is reduced per unit of product.

Although the dilution of maintenance concept describes improvements in individual animal efficiency with increases in milk output, many other factors will influence feed efficiency and the subsequent environmental footprint over an animal’s lifetime and at a farm-system scale. In addition to some of the reproduction and life-cycle management impacts mentioned by [14], feed efficiency can be improved by selecting cows that consume less feed while producing the same amount of milk. Conceptually, this is equivalent to reducing the denominator in the feed efficiency equation, whereas the dilution of maintenance is focused on increasing the numerator. While we have been effective at increasing the numerator increasing the temporal milk production per cow, we have been less effective at improving efficiency by reducing the feed required to produce a given quantity of milk. The work in [14] illustrates this point with the equivalent nutrient requirements for milk production between 2007 and 2017. Both mechanisms for improving feed efficiency (increase in milk production per animal and reduction in feed intake per kg of milk) are influenced by animal genetics and diet composition, among other things. However, the methods for measuring and influencing these mechanisms on-farm are quite different.

Residual feed intake (RFI, kg DMI/cow/d) is a metric used to evaluate differences in feed efficiency between cows with the goal of identifying animals with improved feed efficiency due to reduced feed consumption for an equivalent production. There is a long history of interest in using RFI as the phenotypic expression of feed efficiency to inform breeding programs [17,18,19], but implementation and adoption has been slow, in part because it is more difficult to measure individual animal intake than milk production. New technologies to measure or estimate feed intake and advances in genomic selection methodologies make selecting for improved feed efficiency through reduced RFI [19,20] more accessible. To advance research into, and the adoption of, different methods to improve feed efficiency, it is important to be able to estimate the expected system-wide impacts of the proposed methods.

Whole-farm models like RuFaS can be used to estimate system outcomes to management or biological changes, but most existing models cannot easily represent the impact of a change in RFI. The aggregate or static representation of animal traits and simplifications in the connection between diet and feed consumption, animal life-cycle, and methane and manure production mean that the impact of a reduced individual animal RFI on whole-farm feed consumption and downstream methane and manure production are difficult to include in most farm systems models. We have intentionally designed the Animal Module of RuFaS to enable the investigation into a wide variety of current and future precision management practices that are expected to influence whole-farm efficiency and environmental outcomes [11]. As a result, the RuFaS animal module is easily adapted to address the question of how changes in feed efficiency influence herd and whole-farm environmental outcomes.

The objective of this work is to document the advances in dairy farm simulation modeling made by the Animal Module as part of the RuFaS modeling ecosystem and to demonstrate its utility through a case study of the environmental impacts of improving feed efficiency. Thus, our first objective is to provide detailed documentation of the algorithms that define management, production, and nutrient flows in a dairy herd. For our second objective, we describe and implement methods to add the representation of variable RFI and use this altered model to compare the impact of RFI on feed intake, enteric methane production, and manure production in a simulated herd with 1000 lactating Holstein cows. The future application of the RuFaS model will support scientific investigation and on-farm decision support related to precision-feeding, breeding, and other dairy herd management practices.

## 2. Materials and Methods

The Ruminant Farm System (RuFaS) model consists of four biophysical modules: animal, manure handling, crop + soil, and feed storage (Figure 1). The simulation inputs include time of simulation, herd characteristics, crop characteristics, and other farm decisions. The required inputs follow a tiered file structure that separates inputs that designate the whole-farm and simulation structure from inputs specific to each of the modules with increasing level of detail associated with inputs at lower tiers. The model uses a daily time-step and is programmed in Python, an adaptable and easy-to-read computer programming language.

The model inputs span decisions at the farm, herd, and animal levels (Appendix A). The first set of inputs include the dates of simulation and corresponding weather information. At the farm level, the user can define housing specifications and feeds available for ration formulation and target herd size. At the herd level, the user can specify the breed, reproduction protocols, and lactation curve parameters. Inputs that define animal characteristics include parameters that define the bodyweight distribution, reproductive efficiency, and probability of disease. The model outputs are exported to CSV and graphic images.

**Figure 2:**
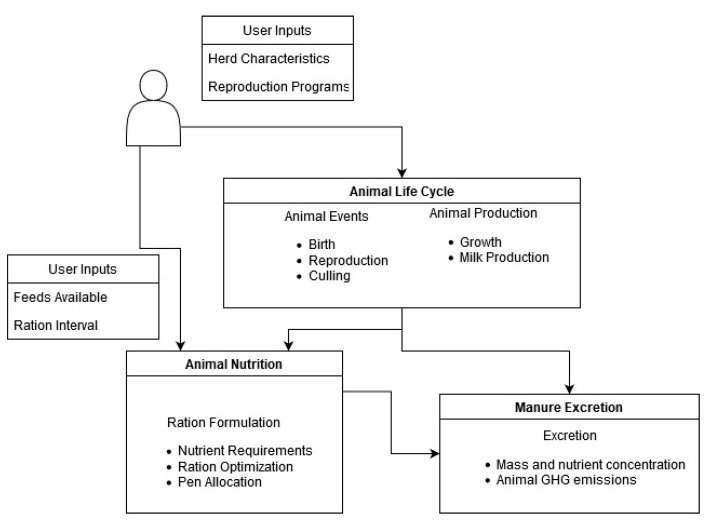
User inputs and main routines of the animal module in the Ruminant Farm Systems model.

The animal module simulates the individual animal from birth to culling, accounting for farm management decisions and individual animal responses to those decisions. Animals are simulated individually, and outputs such as animal growth, animal production, and manure production are estimated daily for each animal. The main routines of the animal module are the animal life cycle, animal nutrition, and manure excretion (Figure 2). The animal management class manages the animal routines—performing the algorithms to account for all animals and animal classes in the simulation.

The animal life cycle simulates the animal from birth to culling, encompassing weaning, first reproductive cycle, first lactation, and subsequent calving and lactation cycles. There are five animal classes: calf, heifer I, heifer II, heifer III, and cow. Calves transition to heifers based on weaning day. The heifer stage is divided into three categories: heifer I (from weaning to breeding period), heifer II (first estrus to transition period), and heifer III (transition period, default one month before calving). Cows include lactating and dry cows. Animals are culled from the herd or added to the herd depending on the management inputs to maintain the herd size specified in the user input.

The life cycle uses stochastic, Monte Carlo methods incorporating the probability of events and parameter averages, distributions, and standard deviations to simulate random variables [21]. The probabilities of each outcome, such as the calf’s sex, conception, or culling, are inputs to the model. Each event’s probability is compared to a randomly generated number between 0 and 1, and, if the probability is greater than the random number, the event occurs. To determine the outcome of the random variables in the model, such as bodyweight, a random number is drawn from a user-defined distribution for that parameter. To prevent extreme values, upper and lower bounds are defined based on recommendations from the literature or biological principles.

Calves are the first animal class initialized, and the success of a calf’s birth, its sex, and its longevity on the farm are determined by stochastic processes as described above. A target average daily gain (ADG) is used to estimate growth until the weaning day is reached. The daily update for the calf includes a check to see if it has reached its weaning day and a method to record bodyweight changes.

The three classes of heifer are used to organize reproduction and life events during the heifer period. Heifer I includes growth until the breeding start day is reached. If timed artificial insemination (TAI) is used, no estrus simulation occurs. The target ADG is set to achieve 55% of mature shrunk bodyweight (BW) at first pregnancy [22]. The heifer II stage incorporates reproductive protocols for conception and pregnancy success. The length of the estrous cycle is determined stochastically, and the simulated day of estrus determines the breeding day. The reproduction programs include timed artificial insemination, estrus detection, and synchronized estrus detection [23]. If the conception was successful (determined stochastically), a pregnancy update determines the gestation length. Stochastic simulation events simulate pregnancy checks at conception and at two other time points to confirm pregnancy. The loss of pregnancy occurs based on a pregnancy loss rate. Pregnancy loss results in an abortion day, and the heifer can be rebred according to rebreeding protocols. Conceptus growth is assumed to be zero prior to 51 days in pregnancy. The total conceptus growth is calculated as:

Total conceptus weight = 0.0148 × gestation length − 2.408 × calf birth weight(1)

Conceptus growth from 51 to the end of gestation is calculated as

Conceptus growth = 3 × (total conceptus weight^1/3^/gestation length − 50)^3^ × (days in pregnancy − 50)^2^ (2)

A heifer moves to the Heifer III class when they are within the user-specified pre-fresh period. Heifers are culled from the herd if they exceed a user-specified heifer reproduction cull time, i.e., if the heifer is not pregnant by the specified age. The target ADG is adjusted to reach 82% of mature shrunk body weight (BW) at the first parturition.

Cows are managed from first calving to culling from the herd. When the days in pregnancy equal the gestation length, the cow calves reset the days in pregnancy to zero and initiate milk production. The milking update estimates milk production from a Wood lactation curve [24,25]. The end of the lactation cycle is a user input, based on the days in pregnancy when the cow is dry. The reproduction program for cows restarts after calving with options for estrus detection, estrus detection with timed artificial insemination, or timed artificial insemination. The start times, pregnancy check times, and success rates of the reproduction protocols are specified by the user. Cows are targeted to grow to their mature body weight by the end of their second lactation. The target average daily gain for cows is set to reach 92% of mature shrunk BW at the end of the first lactation and full mature shrunk body weight by the end of the second lactation. Estimates for lactation-related body weight changes represent the tissue change due to lactation and can be positive or negative. This value is only estimated for lactating cows and is assumed to be 0 for dry cows.

Lactation BW change = −20/65 × exp(1-days in milk/65) + 20/(65^2) × days in milk × exp(1-days in milk/65) if parity = 1 Or −40/70 × exp(1-days in milk/70) + 40/(70^2) × days in milk × exp(1-days in milk/70) if parity > 1(3)

where BW is bodyweight (kg). The daily body weight change of a cow is the sum of the target ADG, conceptus weight change (same as heifer pregnancy), and bodyweight tissues.

The user input herd number is used to maintain the herd size. If the number of heifers is greater than the herd needs, Heifer IIIs will be sold. If the number of heifers is less than the herd needs to maintain the herd size, Heifer IIIs are purchased from the replacement market. Animals can be culled at any life stage depending on user inputs. Male calves can be sold, heifers culled for reproduction failures, and cows for reproduction or milk production issues. Health reasons for culling include lameness, injury, mastitis, disease, udder problems, and unknown issues.

The animal nutrition routine automatically formulates a ration to meet animal nutrient requirements using the feeds specified by user inputs. Feed nutrient composition is based on feed tables in the 2001 NRC with new 2021 starch values added for starch concentrations (Table S1). The maximum feed intake of feeds can be specified by the user, and default settings for certain feeds are set based on the literature.

For calves, the feed intake of milk or milk replacer is assumed to be 10% of the birth weight, a common industry practice (E. Miller-Cushion, personal communication) [26]. The intake of calf starter was estimated from data reported by Khan, M. A., D. M. Weary, and M. A. von Keyserlingk [27] using a broken line regression:

Starter intake (kg) = −0.24783 + 0.0049567 × body weight if body weight ≤ 69.365 Or −6.2263 + 0.091145 × body weight if body weight > 69.365,(4)

where the body weight is in kilograms. During weaning, the length of the weaning period is used to calculate the reduction in milk intake each day. Calf feed intake is used to determine the energy allowable or protein allowable gain. The minimum can be used for determining the daily calf growth as a mechanistic alternative to the ADG estimates set in the life-cycle update, based on the length of the weaning period.

The ration formulation for heifers and cows includes four key processes: requirement calculation, compiling types and amounts of available feeds, nonlinear program optimization, and ration reporting. Rations for heifers and cows are formulated to meet the energy, protein, Ca, and P requirements provided in the NRC [22]. Individual animal requirements are calculated based on animal bodyweight, milk production, growth, and the environment. Individual requirements are then averaged by pen before completing the optimization to generate a ration that meets the average nutrient requirements of each pen. Pen-level summaries of nutrient requirements can be adjusted to formulate diets that meet or exceed the requirements of a larger proportion of the animals within the pen. Net energy, metabolizable protein, and mineral supply are calculated based on the nutrient composition in the feed library and the recommendations in the NRC [22].

Automated ration formulation currently uses an algorithm for least-cost non-linear optimization similar to that described by Rotz [28]; however, we plan to implement additional single and multi-objective optimization methods in future iterations of RuFaS (e.g., goal-programming and multi-objective optimization that include environmental outcomes [29]). In the current version, the optimization routine uses sequential quadratic programming to minimize the price of the ration while meeting nutrient requirements:

Min z_1_ = Σj = 1 c_j_x_j_, x_j_ ≥ 0, (5)

Σ_j = 1_ a_ij_x_j_ ≥ b_i_, i = 1,…, m (6)

where z_1_ is the price of the ration ($), c_j_ is the price of feed j ($/kg), x_j_ is the amount of feed j (kg), and a_ij_ is the nutrient concentration of the feed (Mcal/kg or %). Sequential quadratic programming is a non-linear optimization method capable of handling both discrete and continuous constraints [30]. In addition to the nutrient requirements for net energy, metabolizable protein, Ca, and P as described above, we include constraints for a minimum dietary NDF concentration of 25% (DM basis), maximum NDF concentration of 40%, minimum dietary forage NDF concentration of 19% (DM Basis), and a maximum dietary fat concentration of 7% (DM basis) [31]. If the algorithm reaches the 100-iteration limit and does not satisfy all constraints, we reduce the estimated daily milk production by 0.5 kg and reenter the optimization routine.

The ration formulated for each pen is applied to each animal for the user-defined period between ration formulations, and the total amount of feed consumed by each pen is recorded daily. The manure subroutine calculates the animal manure excretion and sums the manure excretion by both animal class and pen. Total manure (kg as-excreted), total solids (kg DM basis), and methane emissions (g/d) are calculated for each animal and for all animal classes based on animal and dietary characteristics.

For calves, total manure and manure solids are calculated according to animal bodyweight [32]. The average methane emissions from calves reported by Pattanaik, A. K., V. R. B. Sastry, R. C. Katiyar, and M. Lal [33] are used to estimate the methane production from calves:

Methane emis = (0.013 × bw^0.75^ × 4.184)/0.05565, (7)

where methane emis are methane emissions (g/d) and bw is animal bodyweight (kg).

For all classes of heifers, total manure, manure solids, and volatile solids are calculated from ASABE [32] equations. The empirical relationship between methane production (L/d) and dry matter intake described Boadi, D. and K. Wittenberg [34] is used to calculate the methane emissions for heifers.

Manure production and methane emissions of cows are divided into lactating and dry cows. For lactating cows, fecal water, total solids, urine excretion, manure excretion, volatile solids (separated into degradable and nondegradable volatile solids) are calculated according to [35] using dry matter intake as a predictor variable. The user has the option to select from three enteric methane emission calculations: (1) the US animal model described by Niu et al. [36], (2) the Mills et al. [37] Mitsherlich Model 3, or (3) the IPCC [38] Tier 2 model. The Mills et al. [37] and IPCC [38] models were selected to provide a comparison to other models commonly used in the dairy industry and environmental sciences. For instance, the Mills et al. [35] Mitsherlich 3 equation is used in the Cornell Net Carbohydrate and Protein System [39] and Integrated Farm System Model [28]. The dietary gross energy is calculated for the IPCC [38] Tier 2 model according to [31] (Equation (S3)). For dry cows, manure excretion, total solids, and volatile solids are calculated according to ASABE [32]. The methane production from dry cows is calculated according to Mills et al. [35]. The animal model described by Niu et al. [34] includes milking parameters, making that equation a poor choice for calculating dry cow methane production. However, most methane emission models are developed and evaluated with lactating cow data, leaving inexact predictions for dry cows. The IFSM model uses the Mills et al. equation [37] to calculate methane emissions from all cows, and thus that approach was used in this model, but future work to improve the methane emission prediction from dry cows is needed.

### 2.1. Running the Simulation

The user has the option to create a new initialization herd for each simulation. Starting a simulation with an initialization herd substantially reduces the time for the model to stabilize. The initialization herd creates a database of animals that are used to populate the herd and replacement heifer market on day 1 of the simulation. The replacement heifer market is a large number of Heifer III class animal instances that can be selected to meet the herd number targets after cow culling. When the user chooses to create a herd initialization database, a minimum of 1000 calves are created and simulated for a minimum of 5000 days. The initialized herd requires the same user inputs as the simulated herd to create a large herd population with the same characteristics (e.g., breed, BW, etc.) as the animals in the desired simulation. An SQLite database file is created to store the simulated animals at the end of the initialization of the program. The simulated animals represent the five animal classes plus a replacement herd of instances of the Heifer III class (Table 1). Random draws from the initialized database of animals form the herd used for the simulation. The number of calves, heifers, and cows along with the total lactating herd size is a user input.

Each animal is updated daily through the execution of a daily update function that is specific to each animal class. The function updates each animal according to its life cycle functions, calculates the daily manure excretion, and, if it is the end of the ration interval, calculates the animal nutrient requirements, redefines lactating cows’ pen assignments, and creates a new ration. Animals are culled from pens, and new animals are selected from the replacement during the daily update.

### 2.2. Feed Efficiency Case Study

To demonstrate the utility of the RuFaS Animal Module, we developed a method to alter the animal-level feed efficiency and used the model to estimate the expected outcomes of a herd that has been bred for improved feed efficiency. We created a feed-efficiency random-variable (ρ*_i_*) that represents the proportional change in DMI of animal *i*. Because the biological mechanisms that drive feed-efficiency are still not well understood, the objective of the parameter ρ is to modify the cow’s DMI without changing the ration formulation or predicted milk production. During the initialization of each animal, a random draw from the user-defined distribution for this parameter is assigned to that animal’s record. After a ration has been formulated for each pen, when the ration is assigned to each animal’s record, the expected intake for that animal is modified by multiplying the previous model-predicted DMI by ρ*_i_*. The individual animal DMI that has now been adjusted to reflect that animal’s feed efficiency is then used as the input to predict the animal’s enteric methane and manure production. The result is a representation of the RFI phenotype that is commonly measured and used to study feed efficiency.

**Figure 3:**
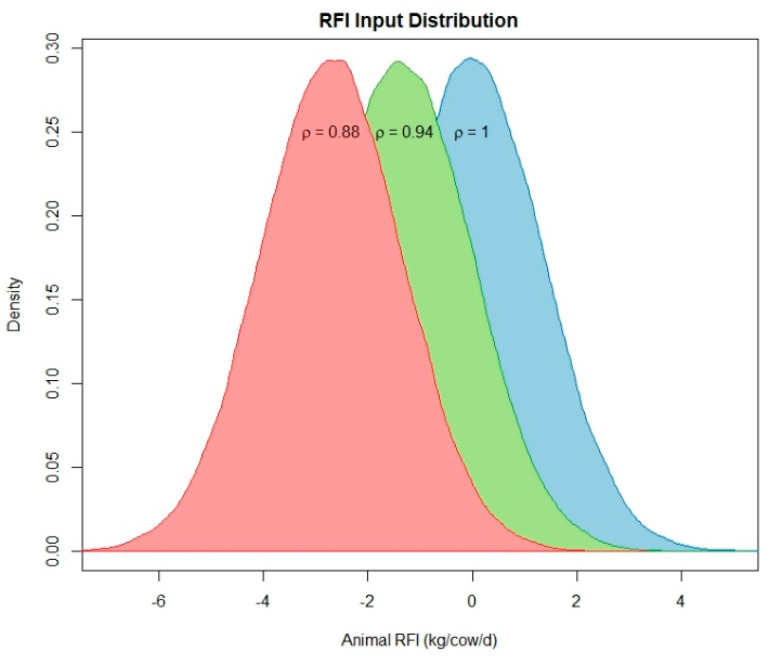
Graphical depiction of the distributions of the ρ parameters implemented to alter the residual feed intake (RFI) which we use as a phenotypic representation of individual animal feed efficiency. ρ represents the proportion of intake.

To illustrate how changes in the distribution of a herd’s RFI are expected to influence environmental outcomes, we simulated herds with 3 different distributions for ρ and compared the predicted outcomes with each other. We based our feed efficiency distributions on recent studies that measured and reported RFI [40]. Based on these studies, we set the standard deviation of the RFI distribution to 6% of the intake for all scenarios and set the mean RFI to 0, −1 SD, and −2 SD, so that the variability in feed efficiency is similar between scenarios but there is a mean shift increasing the mean population efficiency. The ρ distributions for each scenario are shown in Figure 3 and are designed to represent (1) a Baseline scenario in which the herd has an average RFI that is similar to present-day efficiency, (2) a High efficiency scenario in which the herd’s mean efficiency shifted to the 16 percentile of present-day efficiencies, and (3) a Very High efficiency scenario in which the herd’s mean efficiency shifted to the 2.5 percentile of present-day efficiency.

For each scenario, we simulated a 1000-cow Holstein herd for 1 year and held all inputs constant (Table A1, Table A2 and Table A3), with the exception of the input to determine the mean of the distribution of ρ. To support the comparison between scenario runs, we set a common seed for the random number generators so that the outcomes of all Monte-Carlo processes are constant between model runs except where the ρ input distribution was altered.

## 3. Results

### 3.1. Herd Demographics

**Figure 4:**
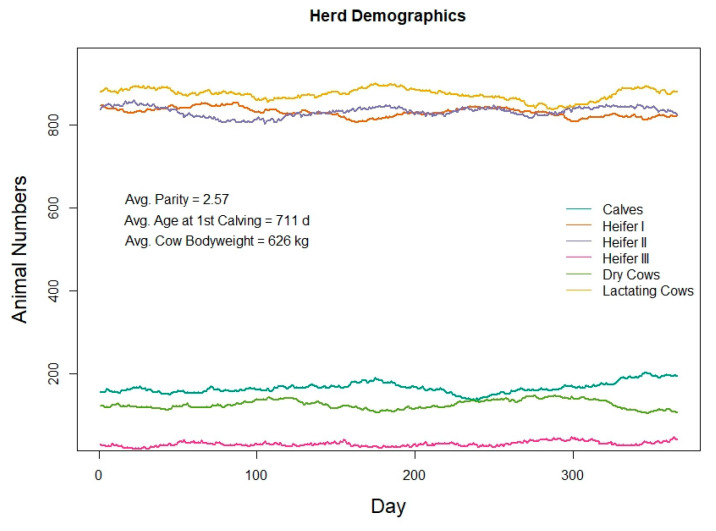
Herd demographics of a 1000-cow Holstein dairy herd simulated in the RuFaS model for 365 days.

As expected, the herd demographic outcomes from all three simulations were the same because the inputs defining the number of animals, grouping management, and reproduction and culling protocols were held constant and the differences in ρ are not expected to influence the animal life-cycle outcomes. The numbers of animals in each animal class (Calves, Heifer I, Heifer II, Heifer II, and Cows divided into lactating cows and dry cows) are shown in Figure 4. This figure demonstrates the capability of the life-cycle model to maintain a consistent number of animals in each animal class across the timeline of the simulation. All female calves were kept in this simulation (A1. “keep female calf rate” = 1), which resulted in a large number of animals in the Heifer I and Heifer II classes. In this situation, the life-cycle algorithms maintain a constant herd size by deciding when to keep a pregnant heifer (Heifer III) or sell her as a replacement animal. During this year of simulation, the model removed 666 heifers from the simulated herd, to be sold as replacement animals. In addition to selling replacements, the life-cycle algorithms simulate the culling of animals for a variety of health and production reasons. The numbers of animals culled for each reason during the 365-day simulation are given in Table 2.

### 3.2. Feed Efficiency Case Study

We successfully implemented a feed efficiency parameter that influences the individual animal intake and reflects the variation in RFI seen in commercial and research herds. The simulated feed efficiency outcomes are given in Table 3 to demonstrate that we achieved the target mean feed efficiencies for the Baseline, High Efficiency, and Very High Efficiency scenarios. The simulated RFI for the Baseline feed efficiency scenario was very close to 0, as desired, whereas the High and Very High Efficiency scenarios had average simulated RFIs of 1.4 and 2.71 kg DM/cow/day, which correspond to a ratio of the simulated DMI:expected DMI of 0.94 and 0.88, respectively. By tracking the simulated intake and comparing it with the expected intake, we confirm that we achieved the desired effect on herd level feed efficiency through the alteration of individual animal outcomes.

**Figure 5:**
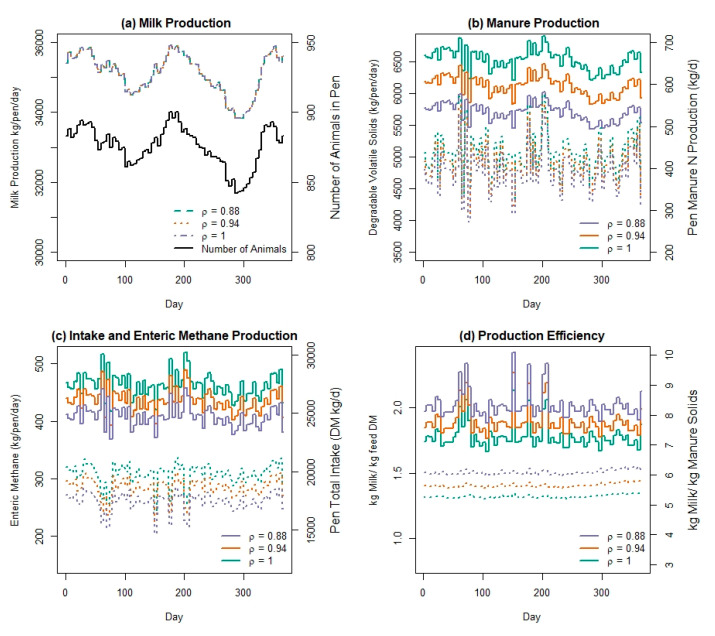
Daily predicted outputs of (**a**) milk production and number of lactating animals, (**b**) manure volatile solids and nitrogen, (**c**) enteric methane and pen intake, and (**d**) production efficiency from a herd of lactating Holstein cows simulated in the Ruminant Farm Systems Animal Module with 3 levels of feed efficiency as implemented by 3 different values of ρ, which is the proportion of expected feed intake consumed for a constant production level. Solid lines: (**b**) Volatile Solids, (**c**) Enteric Methane, (**d**) ratio of Milk:Feed; dotted lines: (**b**) Manure N, (**c**) Intake, (**d**) ratio of Milk:Manure.

The expected impact of feed efficiency on herd production, intake, enteric methane, and manure production is shown in Figure 5. Figure 5a shows that each scenario achieved an equivalent milk production on every day of the simulation, as expected. Panel (a) also highlights oscillations in milk production throughout the year-long simulation, as the total number of lactating animals oscillates with individual animal reproduction and lactation events. We highlight some of the simulated manure characteristics in Figure 5b, and the impact of each feed efficiency scenario on manure outcomes is demonstrated. The total pen DMI shown in Figure 5c represents the daily sum of intake of a ration that is formulated at the user-defined ration interval of 3 days. The composition of the ration at each interval will vary in response to the varying nutrient requirements of the herd at the start of each ration formulation interval. The average and SD of the inclusion rate of each feed are the same for each scenario because the feed-efficiency parameter (ρ) was designed to create a distribution in intake responses to the same formulated ration. Thus, the ration formulation stays the same but the simulated feed consumed is altered. 

The average and SD of the inclusion rates for each feed are given in Table 4. When the animal module is integrated with the rest of the RuFaS simulation platform, the associated GHG emissions and environmental footprint of producing each of these feeds will be included in the model outputs and separated by farm-grown and purchased feeds.

**Figure 6:**
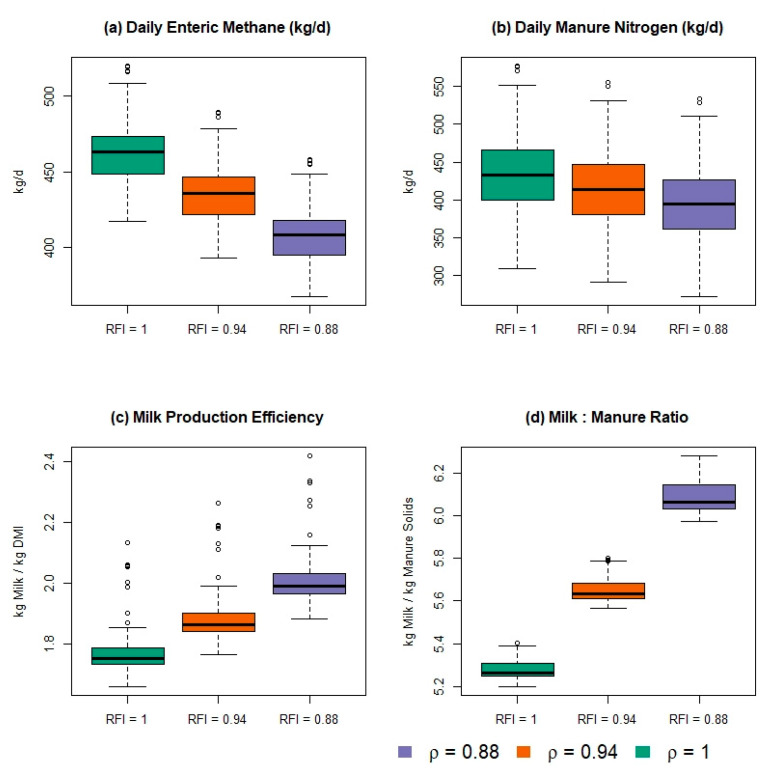
Boxplots of the distribution of daily expected (**a**) enteric methane, (**b**) manure nitrogen, (**c**) milk production efficiency, and (**d**) kg of milk production per kg of total manure solids from a 1000-cow herd of lactating Holstein cows simulated in the Ruminant Farm Systems Animal Module for 365 days with 3 levels of feed efficiency, as implemented by 3 different values of ρ, which is the proportion of expected feed intake consumed for a constant production level.

We display the daily simulated outputs for enteric methane, manure N, milk production efficiency, and milk:manure ratio in box-plots in Figure 6, to facilitate the comparison of the expected distribution of outcomes from each feed efficiency scenario. The variation in outcomes shown in Figure 6 is a reflection of the multiple Monte-Carlo processes used in the Animal Module and the day-to-day variability in the combined number of animals in each stage of lactation. These distributions of responses are more representative of the expected outcomes in a commercial farm setting and provide a better basis for inference and comparison than deterministic mean estimates.

Although the RuFaS Animal Module is not yet integrated with the process-based manure management simulation, we provide an empirical estimate of the carbon footprint of the predicted enteric methane, manure volatile solids, and manure nitrogen using the methods described by IPCC [41]. For manure-based emissions, we used the methods for estimating GHG production from an anaerobic manure lagoon in North America. We chose to use a 100-yr CO_2_-eq of 30 for CH_4_ and a CO_2_-eq of 298 for N_2_O for the purpose of illustrating the comparative impacts [41]. Based on these estimates, the expected decrease in manure excretion from a herd with lower average RFI will result in an even larger reduction in CO_2_-eq than the expected benefit from reduced enteric methane (Table 5).

## 4. Discussion

The Animal Module of RuFaS offers a comprehensive representation of individual dairy cow life events, feeding, milk production, and manure production, as well as the application of a wide variety of herd management options to simulate dynamic, holistic outcomes from the animal part of a dairy farm system. These holistic outcomes and their expected response to different management scenarios are essential information for scientists and industry members to guide sustainable development in the dairy sector. Although the static, deterministic estimates of environmental outcomes offered by LCAs [13,14] are useful signposts, the integration of scientific knowledge through dynamic, stochastic process models provides a tool for comparison across systems, practices, and farm inputs that national averages lack. As a tool for estimating farm-level outcomes, it is essential that RuFaS be both capable of representing commonly used management practices and adaptable to new technologies and practices as they develop. For this reason, we built the RuFaS Animal Module to be flexible in how it represents herd dynamics and included the ability to alter both animal and herd level outcomes in response to simulated changes in management or biology. For example, the representation of reproductive protocols that can be applied separately to heifers and cows enables the model to probe questions about how reproductive efficiency and herd dynamics influence expected environmental outcomes. Although the ration formulation is based on a least-cost optimization, the user can define any number of feeds to be included and set inclusion limits to direct the algorithm’s ration formulation. One more example of built-in flexibility is the housing structure in which the user can define the type of housing, which is similar to other models, but also allows the user to separate the animals into any number of pens and define pen-specific distances to the milking parlor, bedding, and manure management characteristics. All of these model attributes combine to provide a flexible dairy animal and herd simulation model that is more representative of, and adaptable to, the management practices in use today than many existing farm models. Among some of the most comprehensive whole-farm models for US systems (e.g., IFSM [7], COMET-Farm, and Manure-DNDC), RuFaS is the only one that simulates individual animals, and this is a core feature from which much of the Animal Module’s flexibility stems.

The feed efficiency case study highlights the utility of simulating individual animals instead of groups or classes of animals and the adaptability of the model to target specific study questions, which is another key feature of the RuFaS model. A single stochastic parameter to represent the observed animal variation in RFI, and thus the feed efficiency, was easily added to the model code base and facilitated the comparison of multiple outcomes of interest with a single input value. This case study was selected because improving feed efficiency through the use of metrics like RFI has gained increased attention in recent years in the scientific literature [20,40,42] and in industry breeding programs [43,44]. The importance of evaluating the expected outcomes of RFI breeding programs at multiple points in the dairy farm system is highlighted in the model outputs listed in Table 5. The ratio of feed intake between the improved efficiency and baseline scenarios matches the mean input values for the feed efficiency parameter ρ, as intended, but the methane and manure estimates each have their own distribution, that is not centered at the mean of the feed efficiency parameters, as might be expected. This demonstrates that the whole-farm GHG emission reductions from a herd that reduced the feed intake by 6% cannot be assumed to be 6% of each outcome of interest. To estimate the combined impact on the CO_2_-eq from enteric methane and manure production, we summed the CO_2_-eq from Table 4 and took the ratio of the improved feed efficiency scenarios to the baseline scenario. We found that the High efficiency scenario reduced the total CO_2_-eq by 4.6% and the Very High efficiency scenario reduced the total CO_2_-eq by 9.3%. This preliminary assessment of the impact of the improved feed efficiency on enteric and manure GHG emissions can be used to support and set targets for breeding programs. In future work, we plan to add the representation of genetic inheritance between dams and calves to simulate not just the target of breeding programs but the speed with which different breeding and management programs will be expected to get there. The cumulative outcomes and environmental benefits of breeding for improved efficiency over the years can then be estimated.

In addition to adding genetic inheritance, future work to fully integrate the Animal Module with modules that represent manure processing and storage, field management, and crop harvest and storage will complete the dairy farm nutrient cycle to allow for the dynamic feedback of animal management on the whole farm and enable process-based estimates of environmental outcomes both down and upstream from the dairy barn. Upon completing version 1 of the whole-farm model, we will publish the code and underlying documentation in an open-source repository, so that scientists and industry professionals may engage with the model directly. The fully integrated model will have many applications in science to probe questions about the outcomes of single and combinations of management changes. The RuFaS model will also have applications in industry as a tool to estimate whole-farm environmental impacts, and expected changes to the adoption of novel practices, so that producers can meet market and societal demands for improved sustainability.

## 5. Conclusions

We have presented a description of the Animal Module of the Ruminant Farm Systems modeling platform, described its structure and advantages over other existing models, and used the example of feed efficiency to illustrate some of its functionalities and applications. The results of the case study demonstrate the milk, manure, methane, and herd demographic outputs available from the RuFaS Animal Module and provide preliminary estimates of the multiple environmental benefits of improving feed efficiency by reducing RFI. The use of the Animal Module within the larger RuFaS modeling platform will enable an even more comprehensive assessment of how improvements in management and biological performance will affect the environmental impacts of dairy production.

## Figures and Tables

**Table 1:** Example initialization and replacement herd characteristics.

Animal Class	Number of Animals	Mean Age (Days)	Days in Milk
Calves	1323	29	-
Heifer I	6425	208	-
Heifer II	5825	509	-
Heifer III	587	667	-
Cows	16465	1289	182
Replacement Market Number	30426	683	-

**Table 2:** Number of animals that were simulated to be removed from the herd due to culling for each of the listed reasons during a 365-day simulation of a 1000-cow herd in the RuFaS model.

Culling Reason	Low Production	Lameness	Injury	Mastitis	Udder Deformity	Unknown
Number of Animals	69	52	85	62	20	44

**Table 3:** Feed efficiency outcomes from 1000-cow herd simulations with 3 different input values for ρ for the Baseline (ρ = 1), High Efficiency (ρ = 0.94), and Very High Efficiency (ρ = 0.88).

Model Output ^1^	Baseline (ρ = 1)	High Efficiency (−1 SD RFI, ρ = 0.94)	Very High Efficiency (−2 SD RFI, ρ = 0.88)
RFI, kg	0.022	1.4	2.71
SD_RFI_, kg	0.022	0.64	0.127
Ρ_sim_	0.00	0.940	0.880
SD_Ρsim_	0.0093	0.0094	0.0096

^1^ Simulated mean and standard deviations (SD) of feed efficiency outcomes. Residual feed intake (RFI, kg DM/d) is the difference between the simulated intake and the expected intake. Ρ_sim_ is the simulated analog of the model input for the proportional change in dry matter intake (ρ). Ρ_sim_ is calculated as the simulated intake/expected intake and averaged over all lactating cows for the 365-day simulation period.

**Table 4:** Expected average and SD of ration composition and total feed intake from a 1000-cow, 365-day RuFaS simulation with 3 levels of feed efficiency: baseline (ρ = 1), high (ρ = 0.94), and very high (ρ = 0.88).

Feed	Ration Composition	Simulated Intake (Tons/Yr)		
	% DM	(ρ = 1)	(ρ = 0.94)	(ρ = 0.88)
Corn Silage	68.9 (9.79)	4,998	4,703	4403
Soybean Meal	27.2 (5.37)	1,973	1,857	1738
Brewers Grain	3.1 (7.26)	210.7	198.3	185.6
Dicalcium phosphate	0.01 (0.029)	0.708	0.666	0.624
Limestone	0.47 (0.030)	3.41	3.21	3.01

**Table 5:** Expected total milk production, enteric methane, manure volatile solids, and manure N from a 1000-cow, 365-day RuFaS simulation with 3 levels of feed efficiency: baseline (ρ = 1), high (ρ = 0.94), and very high (ρ = 0.88).

Feed Efficiency	Milk (Ton/Yr)	Enteric Methane (Ton/Yr)		Manure Volatile Solids (Ton/Yr)			Manure N (Ton/Yr)	Direct N_2_O from Manure (Ton/Yr)	
-	-	CH_4_	CO_2_-eq	mass	CH_4_	CO_2_-eq	-	N_2_O	CO_2_-eq
Baseline	12,874	169.5	5085	2390	270.0	8099	159.2	0.500	155,110
High Efficiency	12,874	159.6	4787	2240	253.4	7592	152.1	0.478	148,190
Very High Efficiency	12,874	149.4	4482	2088	235.9	7077	144.9	0.455	141,140

## Data Availability

The data presented in this study are available on request from the corresponding author. The data and model code are not currently publicly available because we are still building the other sections of the model.

## Supplementary Materials

The following are available online at https://www.mdpi.com/article/10.3390/ani11051373/s1; Table S1: Feed Library.

## Appendix A. User Inputs for the Animal Module of the Ruminant Farm System

**Table A1:** User inputs in the Ruminant Farm Systems Model related to herd information.

Item	Type	Value	Description
Herd Information			
*Calf No.*	Integer	75	Number of calves randomly selected from initialization herd
*Heifer I No.*	Integer	150	Number of heifers between weaning and first breeding randomly selected from the initialization herd
*Heifer II No.*	Integer	150	Number of heifers between first breeding and close to parturition randomly selected from the initialization herd
*Heifer III No.*	Integer	40	Number of heifers close to parturition randomly selected from the initialization herd
*Cow No.*	Integer	1000	Number of cows randomly selected from the initialization herd
*Replace No.*	Integer	5000	Number of
*Herd No.*	Integer	1000	Goal for number of cows in the herd
*Herd init*	Boolean	False	When herd init is true, simulate a replacement herd database to populate the farm simulation
*Breed*	“HO” or “JE”	“HO”	The breed of cattle in the simulation. Input “HO” for Holsteins and “JE” for Jerseys.
Animal Life Cycle Inputs			
*Breeding start day*	Integer	420	Target start days born of reproduction protocols
*Heifer repro method*	“TAI” other protocols	“TAI”	Reproductive protocol for heifers
*Cow repro method*	protocol	“TAI”	Reproductive protocol for cows
*Semen type*	“conventional” or “sexed”	“conventional”	Type of semen used in reproduction protocols
*Days in preg when dry*	integer	218	Days when the cow is dried off after parturition
*Lactation curve*	“wood” or “milkbot”	“wood”	Model selection for milk production
*Heifer repro cull time*	Integer	650	Days old when a heifer would be culled if unsuccessful in breeding
*Repro cull time*	Integer	300	Threshold of heifer culling age: when the heifer is not pregnant at this age, she will be culled for repro failure
*Do not breed time*	Integer	300	Days in pregnancy when reproduction protocols are stopped: when the cow is not pregnant at this DIM, it will not be bred anymore and will be culled when her milk production drops below the production culling line.
*Cull milk production*	Number	22	Minimum milk production before animal is culled
*Milkings per day*	Integer	1	Number of times per day cows are milked

**Table A2:** User inputs in the Ruminant Farm Systems Model related to animal level management.

Item	Type	Value	Description
*Male calf rate sexed semen*	Decimal	0.1	Probability of male calf if sexed semen used
*Male calf rate conventional semen*	Decimal	0.53	Probability of male calf if sexed semen used
*Birth weight average ho*	Number	43.9	Average birth weight of Holstein cattle
*Birth weight std ho*	Number	1.0	Standard deviation of birth weight of Holstein cattle
*Birth weight average je*	Number	35.2	Average birth weight of Jersey cattle
*Birth weight std je*	Number	1	Standard deviation of birth weight of Jersey cattle
*Keep female calf rate*	Number	1	The rate female calves are kept and raised on-farm
*Wean day*	Integer	60	Day the calf is fully weaned from milk or milk replacer
*Wean length*	Integer	7	Number of days that the cow is stepped down from milk or milk replacer
*Milk type*	“whole” or “replacer”	‘whole’	Type of milk fed to calves
*Grow end day*	Integer	780	Days when animal will cease growing to reach target mature body weight
*Mature body weight, left*	Number	730	The minimum, mode, and maximum values defining the triangular distribution of Mature Body Weight
*Mature body weight, mode*	Number	750	
*Mature body weight, right*	Number	770	

**Table A3:** User inputs in the Ruminant Farm Systems Model related to pen level management.

Item	Type	Value	Description
*Methane model*	“IPCC”, “Mills”, “Niu”	“IPCC”	Methane model for lactating cows
*Ration* *Formulation interval*	Integer	3	Number of days between reformulating animal rations
Pen Characteristics			
*Id*	Integer	-	Pen identification number
*Vertical dist to milking parlor*	Number	0.2	Change in elevation between the pen and milking parlor
*Horizontal dist to milking parlor*	Number	1.6	Flat distance between the pen and milking parlor
*Number of stalls*	Integer	1000	Number of stalls in barn. The number of animals in the pen can be 120% of the number of stalls.
*Bedding type*	“sand” “manure solids” “organics”	“sand”	Type of bedding used in the barn
*Pen type*	“tiestall” or “freestall”	“freestall”	Type of pen
*Manure management*	“default”: “manual scraping” “flush system” “anaerobic lagoon”	“flush”	Options for manure management with handling, separation, treatment, and storage options specified in the manure management inputs.

## References

[1] FAO (2017). The Future of food and Agriculture—Trends and Challenges.

[2] Keyzer M.A., Merbis M., Pavel I., Van Wesenbeeck C. (2005). Diet shifts towards meat and the effects on cereal use: Can we feed the animals in 2030?. Ecol. Econ..

[3] White R.R., Hall M.B. (2017). Nutritional and greenhouse gas impacts of removing animals from US agriculture. Proc. Natl. Acad. Sci. USA.

[4] Marti D., Johnson R.J., Mathews K.H. (2011). Where’s the (Not) Meat?: Byproducts from Beef and Pork Production.

[5] Garnett T. (2013). Food sustainability: Problems, perspectives and solutions. Proc. Nutr. Soc..

[6] Beede D. (2013). 18 Animal Agriculture: How Can It Be Sustainable in the Future?. Sustain. Anim. Agric..

[7] Rotz C., Isenberg B., Stackhouse-Lawson K., Pollak E. (2013). A simulation-based approach for evaluating and comparing the environmental footprints of beef production systems. J. Anim. Sci..

[8] Johnson I., Chapman D., Snow V., Eckard R., Parsons A., Lambert M., Cullen B. (2008). DairyMod and EcoMod: Biophysical pasture-simulation models for Australia and New Zealand. Aust. J. Exp. Agric..

[9] Cabrera V.E., Hildebrand P.E., Jones J.W., Letson D., De Vries A. (2006). An integrated North Florida dairy farm model to reduce environmental impacts under seasonal climate variability. Agric. Ecosyst. Environ..

[10] Del Prado A., Misselbrook T., Chadwick D., Hopkins A., Dewhurst R., Davison P., Butler A., Schröder J., Scholefield D. (2011). SIMSDAIRY: A modelling framework to identify sustainable dairy farms in the UK. Framework description and test for organic systems and N fertiliser optimisation. Sci. Total Environ..

[11] Kebreab E., Reed K.F., Cabrera V.E., Vadas P.A., Thoma G., Tricarico J.M. (2019). A new modeling environment for integrated dairy system management. Anim. Front..

[12] Jones J.W., Antle J.M., Basso B., Boote K.J., Conant R.T., Foster I., Godfray H.C.J., Herrero M., Howitt R.E., Janssen S. (2017). Brief history of agricultural systems modeling. Agric. Syst..

[13] Thoma G., Popp J., Nutter D., Shonnard D., Ulrich R., Matlock M., Kim D.S., Neiderman Z., Kemper N., East C. (2013). Greenhouse gas emissions from milk production and consumption in the United States: A cradle to grave life cycle assessment circa 2008. Int. Dairy J..

[14] Capper J.L., Cady R.A. (2020). The effects of improved performance in the U.S. dairy cattle industry on environmental impacts between 2007 and 2017. J. Anim. Sci..

[15] Capper J.L., Cady R.A., Bauman D.E. (2009). The environmental impact of dairy production: 1944 compared with 2007. J. Anim. Sci..

[16] Capper J.L., Bauman D.E. (2013). The role of productivity in improving the environmental sustainabiliyt of ruminant production systems. Annu. Rev. Anim. Biosci..

[17] Herd R.M., Oddy V.H., Richardson E.C. (2004). Biological basis for variation in residual feed intake in beef cattle. 1. Review of potential mechanisms. Aust. J. Exp. Agric..

[18] Hoque M.A., Susuki K. (2009). Genetics of residual feed intake in cattle and pigs: A Review. Asian-Australas. J. Anim. Sci..

[19] VandeHaar M.J., Armentano L.E., Weigel K., Spurlock D.M., Tempelman R.J., Veerkamp R. (2016). Harnessing the genetics of the modern dairy cow to continue improvements in feed efficiency. J. Dairy Sci..

[20] Li B., VanRaden P.M., Guduk E., O’Connell J.R., Null D.J., Connor E.E., VandeHaar M.J., Tempelman R.J., Weigel K.A., Cole J.B. (2020). Genomic prediction of residual feed intake in US Holstein dairy cattle. J. Dairy Sci..

[21] Rubenstein R.Y., Kroese D.P. (2016). Simulation of Discrete-Event Systems. Simulation and the Monte Carlo Method.

[22] NRC (2001). Nutrient Requirements of Dairy Cattle Seventh Revised Edition.

[23] DCRC (2018). Dairy Reproduction Protocols. Dairy Cattle Reproduction Council. https://www.dcrcouncil.org/protocols/.

[24] Wood P. (1969). Factors affecting the shape of the lactation curve in cattle. Anim. Sci..

[25] Wood P. (1976). Algebraic models of the lactation curves for milk, fat and protein production, with estimates of seasonal variation. Anim. Sci..

[26] Miller-Cushion E. (2019). (University of Florida, Gainesville, FL, USA). Personal communication.

[27] Khan M.A., Weary D.M., von Keyserlingk M.A. (2011). Invited review: Effects of milk ration on solid feed intake, weaning, and performance in dairy heifers. J. Dairy Sci..

[28] Rotz C.A., Corson M.S., Chianese D.S., Montes F., Hafner S.D., Coiner C.U. (2013). Integrated Farm System Model: Reference Manual.

[29] Qu J., Hsiao T., DePeters E., Zaccaria D., Snyder R., Fadel J. (2019). A goal programming approach for balancing diet costs and feed water use under different environmental conditions. J. Dairy Sci..

[30] Boggs P.T., Tolle J.W. (1995). Sequential Quadratic Programming. Acta Numer..

[31] Moraes L.E., Wilen J.E., Robinson P.H., Fadel J.G. (2012). A linear programming model to optimize diets in environmental policy scenarios. J. Dairy Sci..

[32] ASABE (2005). Manure Production and Characteristics.

[33] Pattanaik A.K., Sastry V.R.B., Katiyar R.C., Lal M. (2003). Influence of Grain Processing and Dietary Protein Degradability on Nitrogen Metabolism, Energy Balance and Methane Production in Young Calves. Asian-Australas. J. Anim. Sci..

[34] Boadi D., Wittenberg K. (2002). Methane production from dairy and beef heifers fed forages differing in nutrient density using the sulphur hexafluoride (SF6) tracer gas technique. Can. J. Anim. Sci..

[35] Appuhamy J.A.D.R.N., Moraes L.E., Wagner-Riddle C., Casper D.P., France J., Kebreab E. (2014). Development of mathematical models to predict volume and nutrient composition of fresh manure from lactating Holstein cows. Anim. Prod. Sci..

[36] Niu M., Kebreab E., Hristov A.N., Oh J., Arndt C., Bannink A., Bayat A.R., Brito A.F., Boland T., Casper D. (2018). Prediction of enteric methane production, yield, and intensity in dairy cattle using an intercontinental database. Glob. Chang. Biol..

[37] Mills J.A., Kebreab E., Yates C.M., Crompton L.A., Cammell S.B., Dhanoa M.S., Agnew R.E., France J. (2003). Alternative approaches to predicting methane emissions from dairy cows. J. Anim. Sci..

[38] IPCC (2006). IPCC Guidelines for National Greenhouse Gas Inventories. https://www.ipcc.ch/report/2006-ipcc-guidelines-for-national-greenhouse-gas-inventories/.

[39] Van Amburgh M., Collao-Saenz E., Higgs R., Ross D., Recktenwald E., Raffrenato E., Chase L., Overton T., Mills J., Foskolos A. (2015). The Cornell Net Carbohydrate and Protein System: Updates to the model and evaluation of version 6.5. J. Dairy Sci..

[40] Connor E.E., Hutchison J.L., Van Tassell C.P., Cole J.B. (2019). Defining the optimal period length and stage of growth or lactation to estimate residual feed intake in dairy cows. J. Dairy Sci..

[41] IPCC Climate Change and Land: An IPCC Special Report on Climate Change, Desertification, Land Degradation, Sustainable Land Management, Food Security, and Greenhouse Gas Fluxes in Terrestrial Ecosystems; 2019. https://www.ipcc.ch/srccl/.

[42] Liu E., VandeHaar M.J. (2020). Relationship of residual feed intake and protein efficiency in lactating cows fed high- or low-protein diets. J. Dairy Sci..

[43] EcoFeed by STI Genetics. https://stgen.com/article/article.aspx?code=4247&language=english&pego=consulta.

[44] Holstein Association USA, I. TPI Formula—April 2021. https://www.holsteinusa.com/genetic_evaluations/ss_tpi_formula.html#.

